# Supplementary material for: Antiviral Activity of Chitosan Nanoparticles and Chitosan Silver Nanocomposites against Alfalfa Mosaic Virus
Source: Polymers (Basel). 2023 Jul 6;15(13):2961. doi: 10.3390/polym15132961 (PMC10346731; doi:10.3390/polym15132961)
Supplement: Supplementary file 1 [file polymers-15-02961-s001.zip › polymers-2431668-supplementary.pdf]

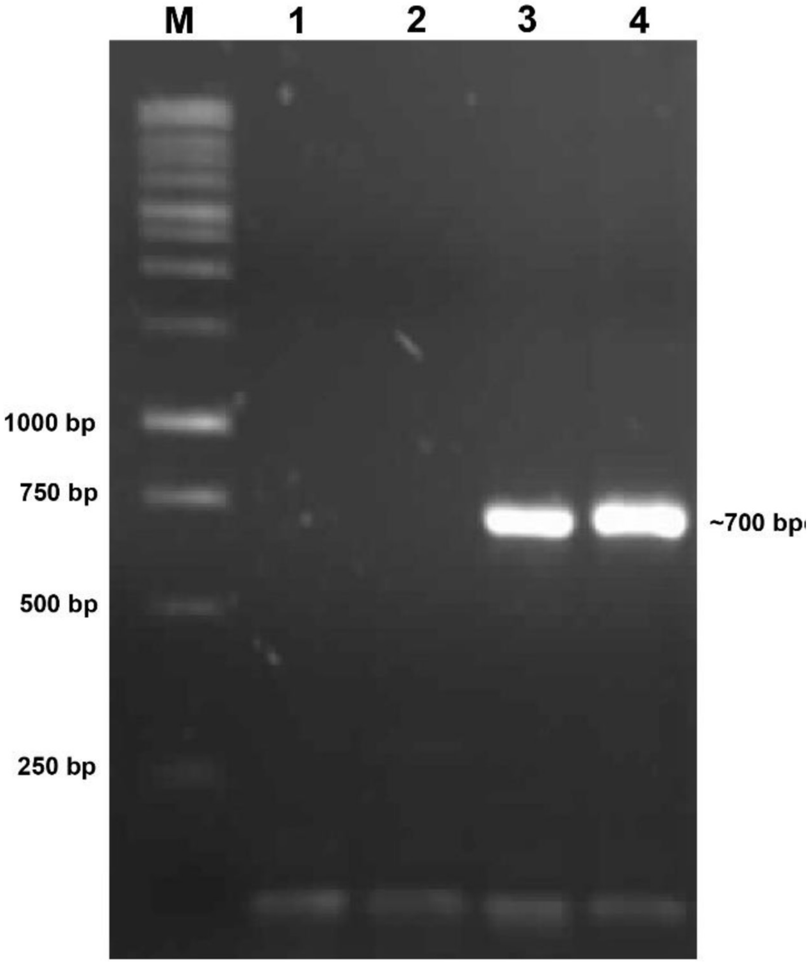

Figure S1. RT-PCR amplified product analysis using agarose gel electrophoresis. M: 1 kb DNA ladder; Lanes 1-2 healthy plant control; Lane 3 pepper plant naturally infected with AMV; Lane 4 pepper plant mechanically inoculated with AMV.

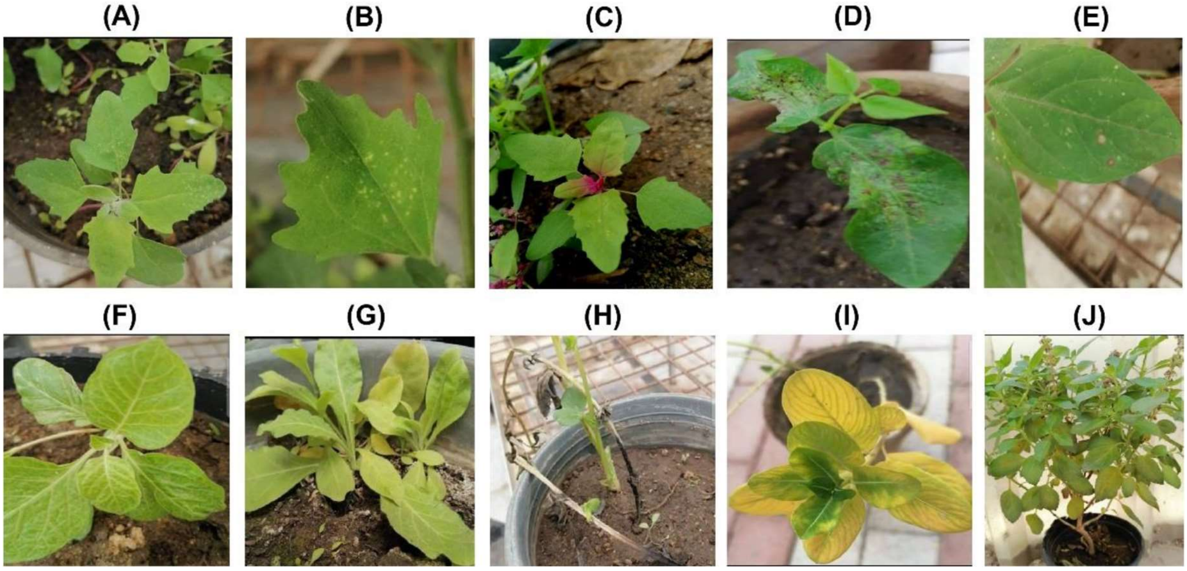

Figure S2. AMV infection symptoms on some differential host plants after mechanical inoculation (**A**): chlorotic local lesions on *Chenopodium quinoa* , (**B**): chlorotic local lesions on *C. murale*, (**C**): chlorotic local lesions on *C amaranticolor*, (**D**): necrotic local lesions on *Vigna unguculata*, (**E**): necrotic local

lesion and chlorotic flecking on *Phaseolus vulgaris*, (**F**): systemic mosaic on *Datura stramonium*, (**G**): systemic mosaic on *Nicotiana tabacum*, (**H**): systemic necrosis and death on *Vicia faba*, (**I**): systemic mosaic and yellowing on *Catharanthus roseus*, (**J**): systemic mosaic and yellowing on *Ocimum basilicum*.
