# Supplementary figures and images for: Antiviral Activity of Chitosan Nanoparticles and Chitosan Silver Nanocomposites against Alfalfa Mosaic Virus
Source: Polymers (Basel). 2023 Jul 6;15(13):2961. doi: 10.3390/polym15132961 (PMC10346731; doi:10.3390/polym15132961)

## Slide 1
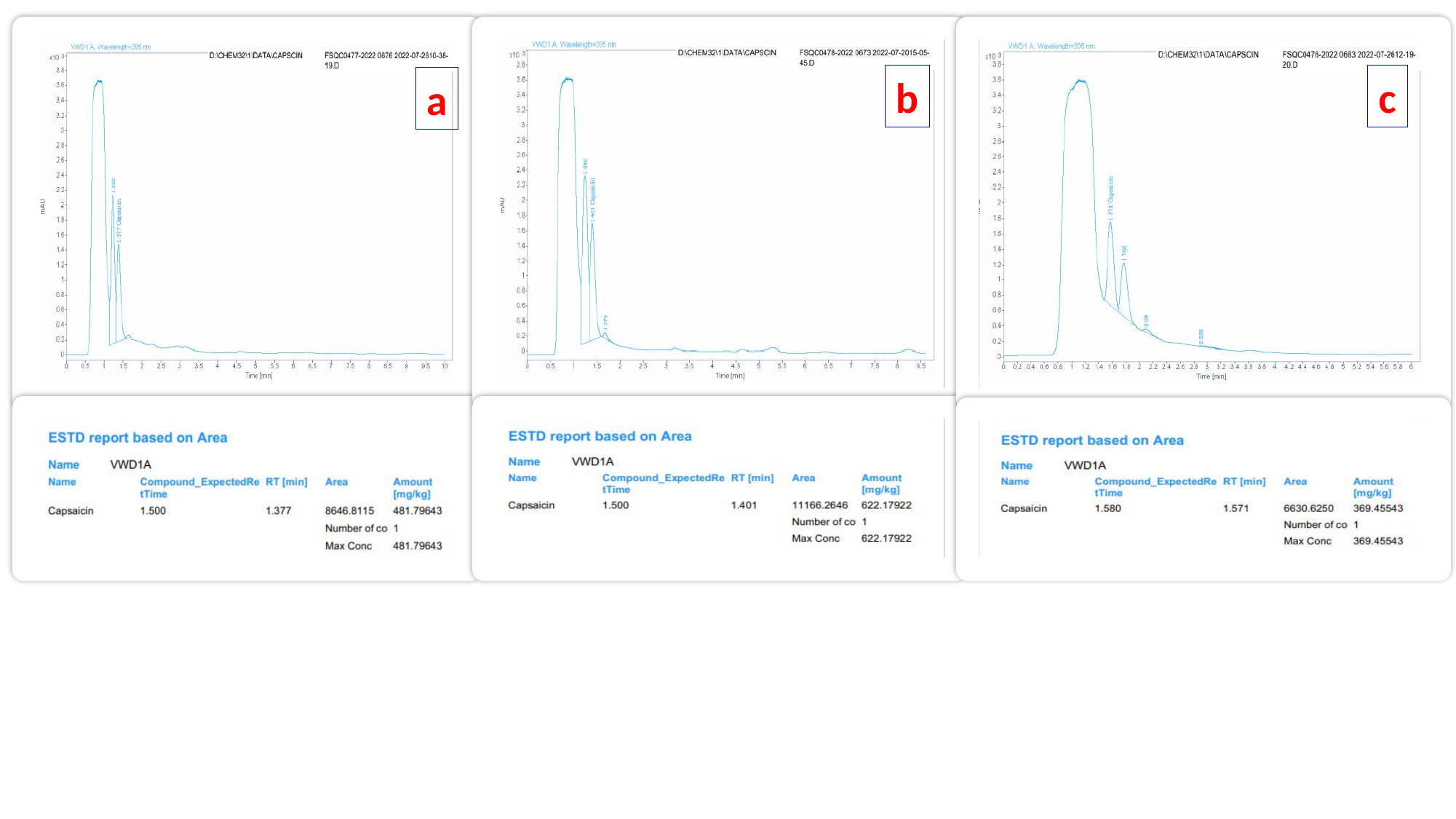

M
b
c
a

Supplement: Supplementary file 1 [file polymers-15-02961-s001.zip › polymers-2431668-supplementary.pptx]
